# Impacts of the 2023 Marine Heatwave in the Florida Keys: Detection and Analysis of a Mass Coral Bleaching Event Using Spaceborne Remote Sensing Imagery

**DOI:** 10.1021/acs.est.5c03122

**Published:** 2025-07-21

**Authors:** Mariam Ayad, Christine M. Lee, James W. Porter, Ved Chirayath, Camilla L. Nivison, Kelsey M. Vaughn, Raphael Kudela

**Affiliations:** † Department of Ocean Sciences, 8787 University of California, Santa Cruz, Santa Cruz, California 95064, United States; ‡ Jet Propulsion Laboratory, 53411 California Institute of Technology, Pasadena, California 91011, United States; § Odum School of Ecology, 1355 University of Georgia, Athens, Georgia 30602, United States; ∥ Rosenstiel School of Marine, Atmospheric, and Earth Science, University of Miami, Miami, Florida 33149, United States

**Keywords:** coral bleaching, coral reef, remote sensing, planet SuperDove, Florida Keys, change detection

## Abstract

Coral reefs are facing several stressors, such as increases in sea surface temperature, eutrophication, and hurricanes, resulting in reef-decline worldwide. In the Florida Keys, these stressors, especially elevated temperatures, have triggered widespread coral bleaching as well as a cascade of simultaneous negative impacts, such as increased disease, accelerated reef erosion, and severe ecosystem degradation. In the summer of 2023, the Florida Keys and the Caribbean experienced a mass bleaching event due to a record-breaking marine heatwave with ocean temperatures exceeding 38 °C. This study investigates whether remote sensing using Planet’s SuperDove sensor could detect this mass coral bleaching event at Horseshoe Reef and Cheeca Rocks in the Florida Keys. We validated these data using several sources: NOAA photomosaic data, NASA airborne fluid lensing from two campaigns (before and during bleaching), and underwater orthomosaic data from July 2023. We were able to detect a signal change using the SuperDove sensor between healthy and bleached coral. Bleached corals have a higher reflectance in SuperDove’s band 2 (492 nm) compared to healthy coral. The results of this study supports the use of Planet’s SuperDove satellite imagery for long-term monitoring of coral bleaching, though confirmation with high-resolution refraction-free data are still needed.

## Graphical abstract

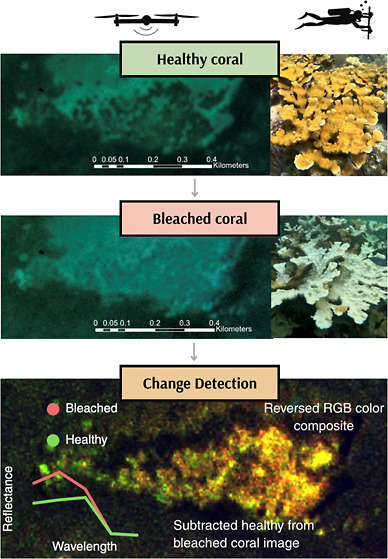

## Introduction

The Florida Keys provide critical ecosystem services to several threatened and endangered marine species and are a key contributor to Florida’s gross domestic product.^1^ Coral reefs in the Florida Keys generate $8.5 billion annually and create 70,400 jobs in the south Florida economy.^2^ Additionally, approximately half of all federally regulated fisheries rely on coral reefs. NOAA’s National Marine Fisheries Service estimates coral reefs contribute over $100 million to the commercial fishing industry annually.^2^ Although coral reefs play a vital role in maintaining a healthy economy, they are one of the most vulnerable ecosystems in a changing climate. Coral reefs face many stressors, such as nutrient pollution, overfishing, sedimentation, increased sea surface temperatures (SST), ocean acidification, oil pollution, terrestrial runoff, physical damage to the coral (from boats, tourists, dredging, and storms), and introduced species. These stressors have resulted in a loss of roughly 40% of coral cover in the Florida Keys in the past 40 years.^3–5^

One of the main causes of coral cover loss and mass coral bleaching events is the increasing frequency and severity of marine heatwaves (MHW).^6^ In 2023, the Florida Keys and the Caribbean experienced record-breaking SST (exceeding 38 °C) (Figure 1), driven by El Niño-Southern Oscillation (ENSO) and amplified by climate change.^7^ This MHW event resulted in a mass coral bleaching event throughout the Florida Keys and Caribbean.^7,8^ Coral bleaching is a stress-induced phenomenon in which corals expel or consume their symbiotic algae, . This process causes corals to turn white, as the absence of exposes the underlying white calcium carbonate skeleton.^9^ The loss of these vital symbionts eliminates the coral’s photosynthetic ability, and thus, its main carbon source, leading to declines in health and potential mortality. However, coral bleaching can be reversed if the stressor, in this case, SST, returns to normal conditions and the corals are recolonized by before they suffer too great an energy deficit.

**Figure 1:**
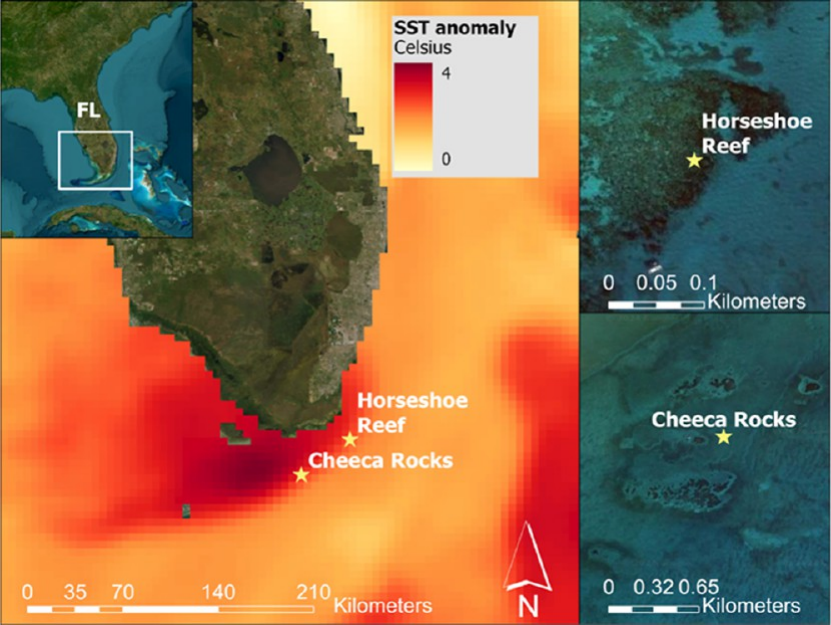
A map of the Florida Keys and the SST anomaly maximum on July 13, 2023 (left panel) with the 2 study sites: Horseshoe Reef and Cheeca Rocks (right panel). The data are the Daily Global 5 km Satellite Sea Surface Temperature Anomaly from NOAA (https://coralreefwatch.noaa.gov/product/5km/).

To effectively manage and protect coral reefs, monitoring and understanding the scope of negative impacts across both spatial and temporal dimensions is essential. Rapid identification of which coral reefs bleach and which are spared will be especially valuable in targeting regions for special protection and restoration efforts. Achieving this requires fine-scale monitoring of reef systems over extensive areas, the scope of which can only be achieved using remote sensing. Remote sensing is a powerful tool for monitoring shallow coral reef habitats because it can rapidly capture large areas compared to labor-intensive diving surveys. Previous studies have indicated that mass coral bleaching events are detectable from satellite data.^10–13^ These studies have primarily focused on the Great Barrier Reef, Australia, because of the availability of extensive survey field data and high coral cover. However, a few studies have investigated other regions, including Hawaii,^14^ China,^15^ Honduras^16^ and the Northwestern Hawaiian Islands and the Gilbert Islands.^17^ These methods require clear imagery, a sensor with frequent revisit times, and high spatial resolution (pixels ≤ 5 m).^10^ In addition, previous studies have found that the detection of coral bleaching using satellite remote sensing is limited to shallow coral reefs with a depth of less than 15 m, as the signal rapidly attenuates with increasing depth.^11,12,16,36^ . Detection of bleached coral can be challenging due to similar spectral signatures of physically adjacent bright targets (e.g., sand or carbonate rock). Furthermore, in cases of overlapping coral types in the same pixel, the reflectance of bleach, live, or algal-covered coral is not readily distinguishable. These challenges highlight the need for a high-resolution sensor, especially for detecting bleaching on reefs with low coral cover.

Coral bleaching can occur on a time scale of weeks; therefore the PlanetScope SuperDove, with its 1 day revisit time was the best sensor for this work. This sensor has a spatial resolution of 3 m/pixel allowing it to record bleaching of very large individual coral heads and clusters of smaller colonies. Previous work by^14^ used Planet Dove imagery to assess large-scale bleaching across the Hawaiian Islands. Their approach estimated bottom reflectance through radiative transfer modeling and implemented a bleaching probability index informed by NOAA Coral Reef Watch (CRW) SST data to define the timing of the bleaching event. While this method provides a valuable framework for regional-scale assessments, it requires inputs such as high-resolution airborne maps of live coral cover, bathymetry, and water optical properties, which are not widely available for many reef systems. In contrast, our study focuses on localized bleaching impacts on two reefs in the Florida Keys, Horseshoe Reef and Cheeca Rocks, during the unprecedented 2023 marine heatwave. We apply a pseudoinvariant feature (PIF) normalization technique to remote sensing reflectance time series, allowing us to detect spectral anomalies associated with bleaching without the need for bottom reflectance modeling or SST-based thresholds. However, this approach requires well-georeferenced validation data of bleached coral cover to confirm the satellite-detected signals. In our case, the 2023 mass bleaching event provided a unique opportunity to test real-time remote sensing, as reports confirmed nearly 100% bleaching across all coral colonies in Florida’s reefs.^18^ This large-scale bleaching event prompted a widespread response, with several research groups and monitoring programs conducting fieldwork to collect in situ data across affected reef sites.

In this study, we combined satellite remote sensing observations with both airborne and diver-based methods to validate bleaching detection. We contributed to this effort by collecting NASA airborne fluid lensing data and in situ orthomosaics of benthic images taken underwater.^19^ Airborne fluid lensing is a NASA-patented technology that produces multispectral orthorectified and refraction-corrected imagery, bathymetry, and 3D models.^20,21^ The FluidCam data set has allowed us to make much broader spatial scale assessments on the extent of bleaching, as compared to diver-based methods.^22,23^ In contrast, diver-generated orthomosaics offered precise, colony-level validation at smaller, patchier sites like Horseshoe Reef. At Cheeca Rocks, we were also able to incorporate publicly available aerial data from NOAA, highlighting how this approach can be adapted for use at other sites where baseline data already exist. If validated, remote-sensing data can provide coastal zone managers and policymakers with inexpensive and effective ways to monitor these valuable and vulnerable ecosystems.^24^

## Data and Methods

### Study Sites

We focus on two study sites:^1^ Horseshoe Reef (total area of 35,029 m^2^) which has sparse coral cover and^2^ Cheeca Rocks (total area of 82,759 m^2^) with dense coral cover (Figure 1 right panel). These patch reefs are part of the Florida Keys National Marine Sanctuary (NMS) which protects 3800 square miles of coastal waters. Horseshoe Reef is a shallow reef (<4 m) located about 7 km offshore and is known for its extensive stands of branching corals, and . is currently threatened by several environmental stressors.^25,26^ Cheeca Rocks is a shallow reef (<6 m) located about 2 km offshore and has dense coral cover of boulder and star corals. This site has exhibited resilience to environmental stressors and has been used by many researchers studying climate change impacts (https://floridakeys.noaa.gov). Given their importance to the protection of coral biodiversity and promotion of commercial activities in the Florida Keys, both Horseshoe Reef and Cheeca Rocks have been designated as NOAA Iconic Reefs.^27–29^ Fieldwork was completed before (May 2023) and during (July-August 2023) this MHW event at Horseshoe Reef (https://earthobservatory.nasa.gov/images/153304/confronting-floridas-coral-collapse).

### Spaceborne Remote Sensing Methods

#### PlanetScope SuperDove Imagery

PlanetScope is the constellation of Earth observation satellites operated by Planet Laboratories Inc., which includes three types of Dove sensors: Dove Classic, Dove-R, and SuperDove. The workflow for preprocessing the remote sensing images is shown in Figure 2. Information on the Planet SuperDove sensor is shown in Table 1. The SuperDove imagery is available from Planet.com and has a revisit time of 1 day and a high spatial resolution of 3 m. All images were carefully selected manually to be free of surface-reflectance glint and have less than 15% cloud cover. For Horseshoe Reef, 16 images were selected and are summarized in Table S1. We selected 2 images from 2022 because they have similar sun elevation to the 2023 bleaching period. We selected approximately 1 image per month from January 2023 to April 2024. Some months were excluded due to rain or wind events that caused increased turbidity, which would impair image quality and comparison.^30^ We used Figure 3 to manually select bleached-coral locations. We calculated the average reflectance over this area. All images were normalized using the April 18, 2023, image. For Cheeca Rocks, 16 images were chosen using similar selection criteria (Table S2). All images were normalized using the May 20, 2023 image.

**Figure 2:**
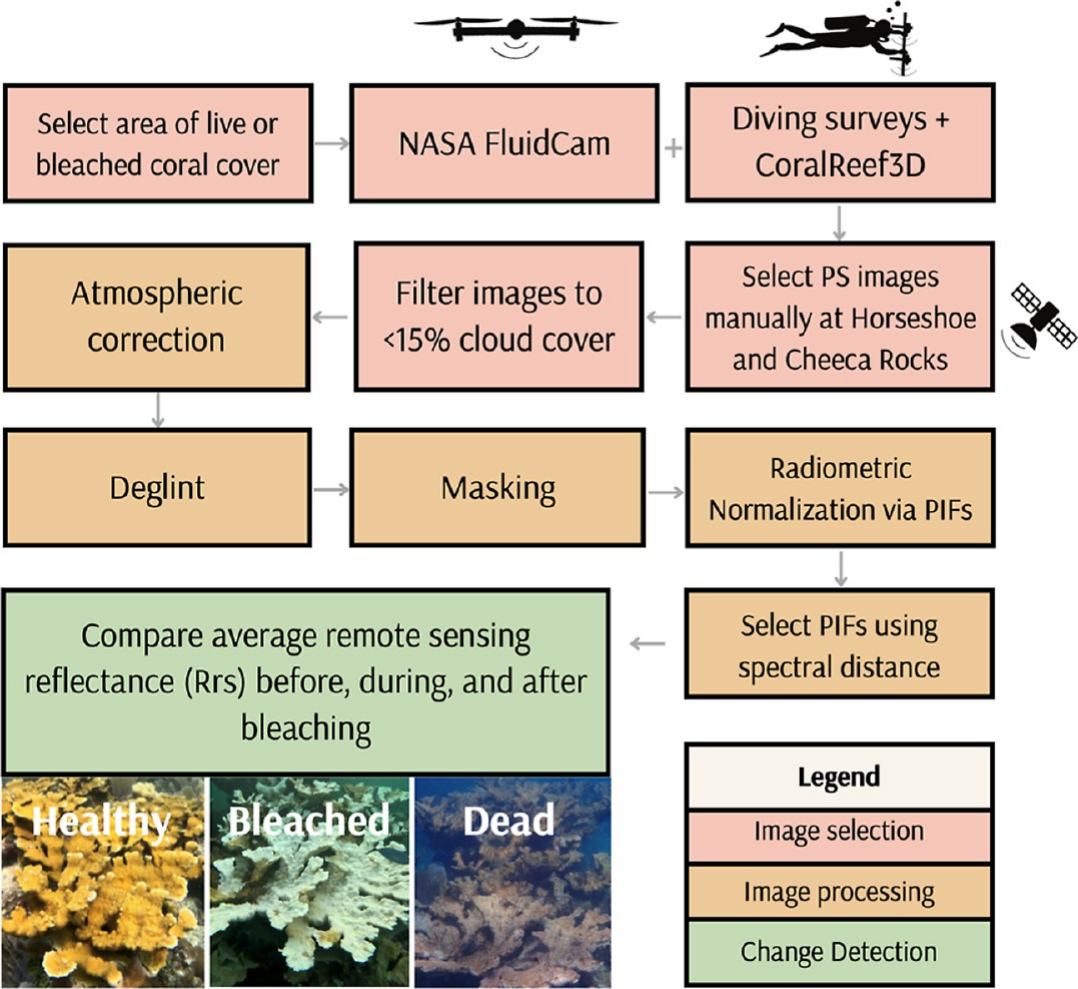
Workflow of satellite remote sensing methods for bleaching detection. Photograph of a dead colony at Horseshoe Reef (taken on May 10, 2024) courtesy of Andrew Ibarra, NOAA Office of National Marine Sanctuaries.

**Table 1:** Sensor Specifications for PlanetScope

sensor	PlanetScope SuperDove (PSB.SD)
revisit time (days)	1
spatial resolution (meters)	3
spectral bands (bands used in this study)	band 1:444 nm: coastal blue
	band 2:492 nm: blue
	band 3:566 nm: green
	band 4:666 nm: red
	band 8:866 nm: NIR

**Figure 3:**
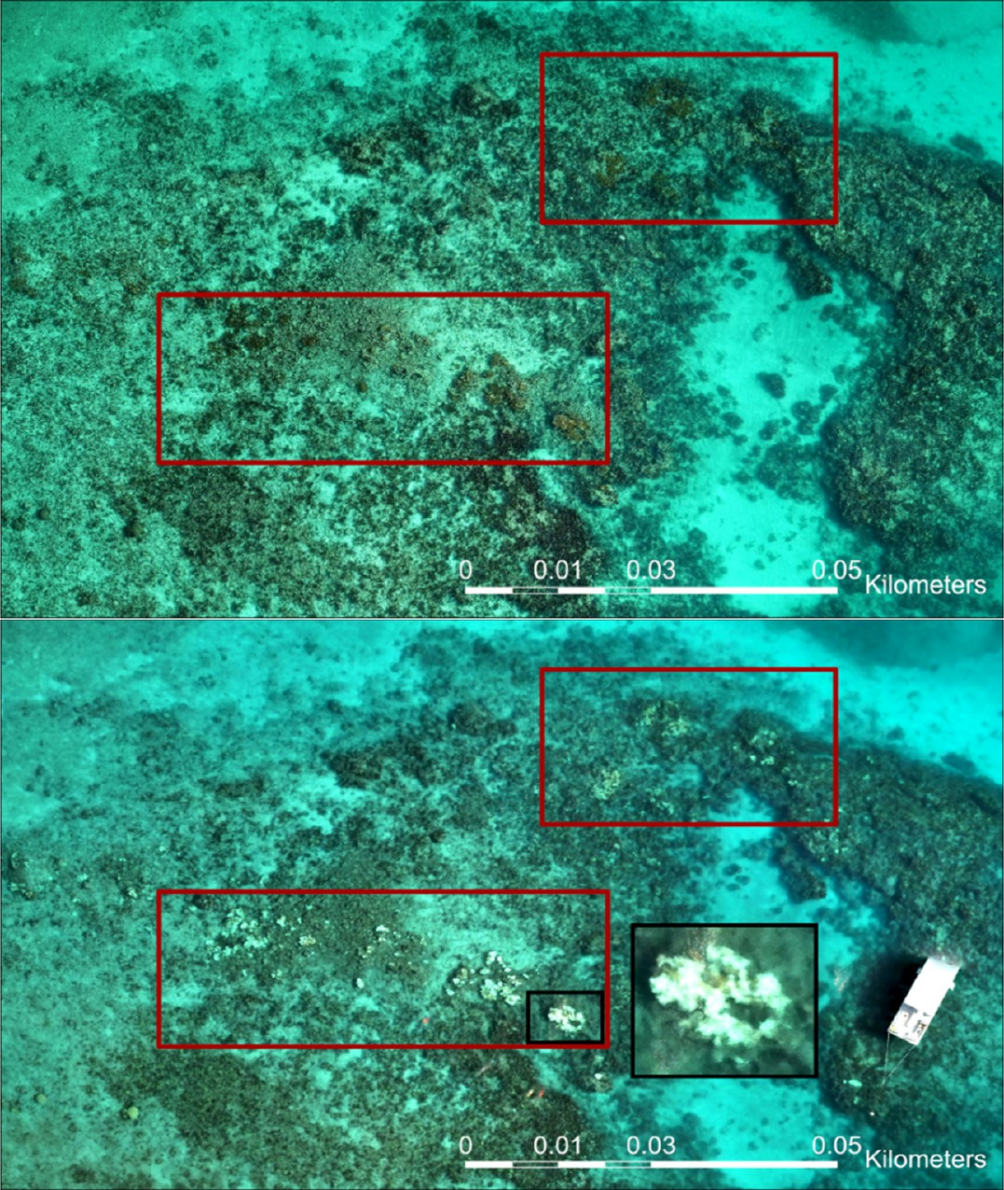
Horseshoe Reef before and during the bleaching event. Airborne Fluid Lensing data set from 5/18/23 (top) and 8/8/2023 (bottom). Red boxes highlight where there is live coral cover. The black box highlights a bleached coral colony.

### Image Processing

ACOLITE is a software tool used to process remote sensing imagery for coastal applications. The software can be downloaded from the GitHub repository: https://github.com/acolite/acolite. The Dark Spectrum Fitting (DSF) algorithm^31,32^ is the atmospheric correction applied to the SuperDove sensor using the ACOLITE software. This DSF algorithm estimates the remote sensing reflectance [*R*_rs_ (sr^–1^)] which is the output for all the images. After applying atmospheric correction, we applied a clear water (low-turbidity) sun glint correction using the NIR band.^33^ A mask was applied to remove land, boats, and clouds from each image.

### Radiometric Normalization Using Pseudo Invariant Features (PIFs)

To compare between remote sensing images, we normalized any distortions in the imagery (caused by atmospheric conditions, view angle, solar angle, etc.) by applying radiometric normalization via Pseudo Invariant Features (PIFs).^34^ PIFs are regions where reflectance is expected to be stable over time. For example, deep water regions, sand regions, algal-dominated environments, and airport/building roofs have served as reference targets in previous studies for coral bleaching detection.^11,15,16,34,35^ This normalization method uses a linear regression of the PIF spectra to determine the transformation values (gain and offset) from the adjusted image to the reference image. These values are applied to normalize the adjusted image to match the reference image. We calculated the spectral distance between the reference and the adjusted image to assist in selecting the PIFs. Spectral distance was calculated using spectral information divergence (SID) to measure the difference between the spectral characteristics of pixels in the reference and adjusted imagery. For Horseshoe and Cheeca Rocks we selected roughly 10–11 PIFs with a size of 30 × 30 pixels. After applying the spectral difference between the adjusted (normalized) image and the reference image, the most stable pixels were found in deep water, shallow-water algal habitats, and benthic sand patches. For Horseshoe Reef, we selected algal habitats and sand pixels; for Cheeca Rocks, we selected deep-water and sand pixels. Increasing the polygon size from 5 × 5 pixels to 100 × 100 pixels results in a reduction of variance in average reflectance values.^35^ We identified that a polygon size of 30 × 30 pixels combined with 10–11 Pseudo-Invariant Features (PIFs) produced the optimal correspondence between the normalized and reference images. Although smaller polygon sizes (e.g., 5 × 5 pixels and 10 × 10 pixels) were initially considered to preserve spatial resolution, no significant improvements were observed below the 30 × 30 size with 10–11 PIFs.

### Horseshoe Reef Data

#### Airborne Fluid Lensing Campaign 2023 Data

Novel NASA airborne fluid lensing was used to image Horseshoe Reef in 3D before^5–18^ and during (8-8-2023) the unprecedented MHW of 2023. Figure 4 shows an example of mm-scale airborne fluid lensing reconstruction of a test target and coral reef with enhanced signal-to-noise ratio (SNR) and effective spatial resolution. The airborne fluid lensing data set was used to capture coral colony-level bleaching over the entirety of Horseshoe Reef for NMS and NOAA (Figure 3).

**Figure 4:**
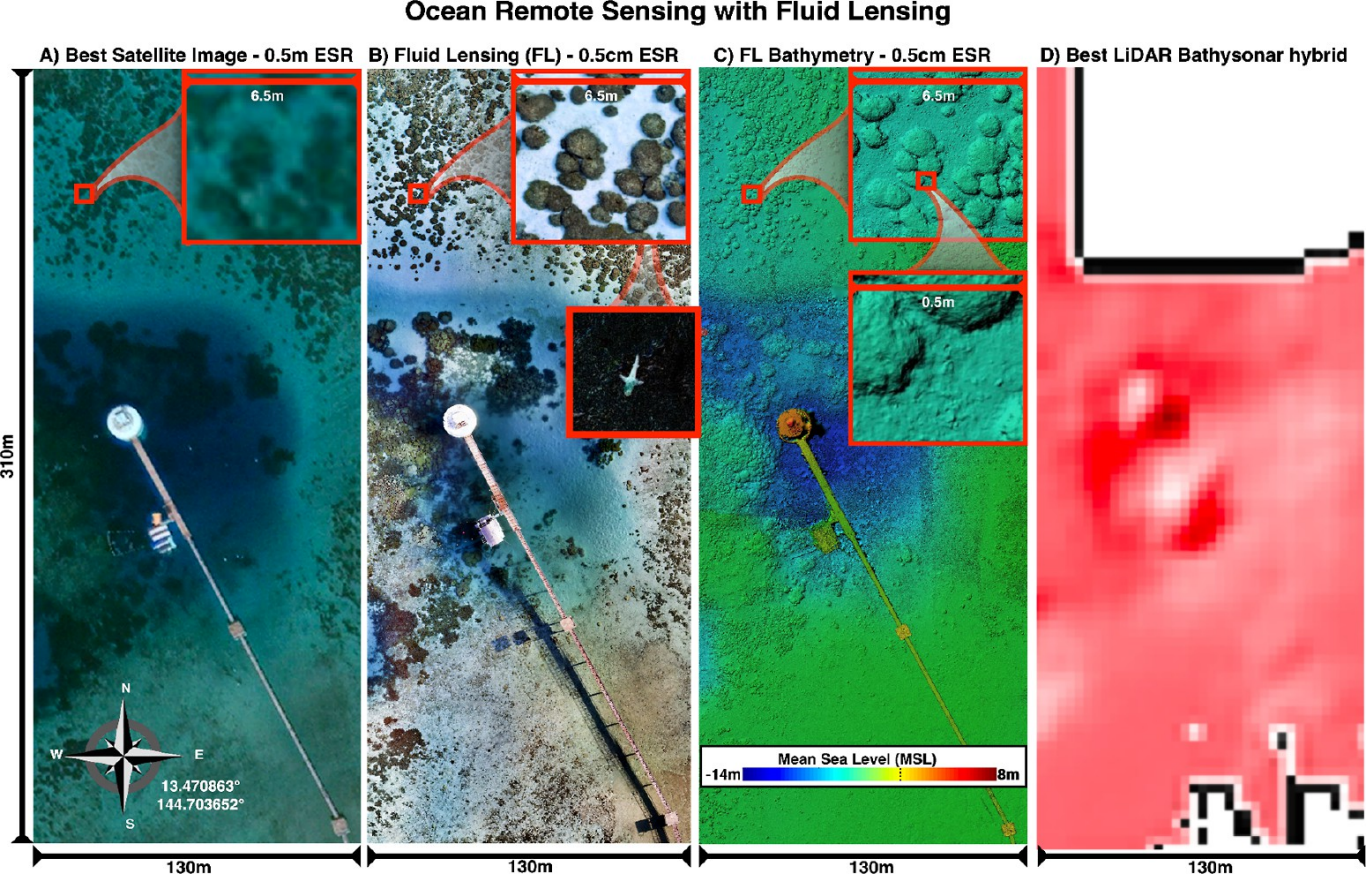
Next-Generation Ocean Remote Sensing with Fluid Lensing. Novel technologies such as fluid lensing permit multispectral 3D benthic imaging without ocean wave distortion and caustic noise. This results in improved signal-to-noise ratio (SNR), effective spatial resolution (ESR), and greater imaging depth. A sample data set from the 2021 airborne Guam 2021 campaign demonstrates the technology’s capability to map complex shallow reef structures with high fidelity. Fluid lensing can also be adapted for use in various fluid environments, such as the hydrocarbon seas on Saturn’s moon Titan. Additionally, it can be applied to imaging through refractive distortions, like those near active deep sea hydrothermal vents on oceans across the solar system.

### Underwater Photogrammetry

Photographs were taken by divers using paired GoPro underwater cameras (Figure 5) with the image-capture rate set automatically at one-second intervals. These orthomosaics provided high-resolution data and facilitated species identifications and accurate assessments of coral health. The images captured on July 11-26, 2023 ^19^ allowed us to ground-truth our airborne fluid lensing images.

**Figure 5:**
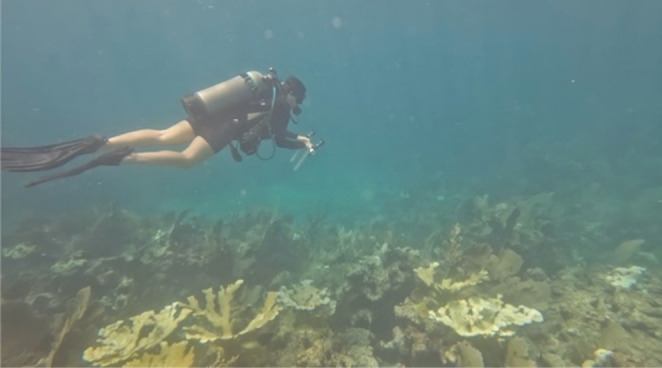
Horseshoe Reef. Diver taking photos of Horseshoe Reef for 3D image analysis and remote sensing validation.

### Cheeca Rocks Data

For Cheeca Rocks, data were acquired from NOAA through reports and photomosaics provided by the National Coral Reef Monitoring Program (https://coris.noaa.gov/monitoring/). Cheeca Rocks had reports of 100% bleaching during the 2023 marine heatwave (https://aoml.noaa.gov/cheeca-rocks-reef-completely-bleached/). We also used airborne imagery collected from NOAA to map live coral cover over Cheeca Rocks. This orthoimage data set is a 4-band mosaic from Miami to Key West and has a spatial resolution of 0.3 m. These were collected in the Winter (1/8) and Spring (5/20) of 2019 (https://fisheries.noaa.gov/inport/item/63292). The data were imported into ArcGIS Pro, where the Iso Cluster Unsupervised Classification tool was utilized to identify the general area of live coral cover. This tool employs the Iso Cluster Unsupervised Classification algorithm, which groups pixels into clusters based on similar spectral characteristics within the imagery. The resulting classification delineates areas of live coral cover, enabling the determination of the average reflectance over these regions across all images.

## Results and Discussion

### Horseshoe Reef Results

The average normalized remote sensing reflectance (sr^–1^) before, during, and after the bleaching event from August 2022 to April 2024 is shown in Figure 6A. The results show that the bleached period (red and orange lines in Figure 6A) has the highest reflectance in bands 1–2. We see a clear separation in reflectance values in band 2 (blue band—492 nm). Figure 6A also includes the data from August 17 and 31, 2022, as represented by the dark gray lines, due to the similar solar angles observed on those dates. However, it remains unclear from this method whether coral paling or bleaching occurred during this period in August 2022.

**Figure 6:**
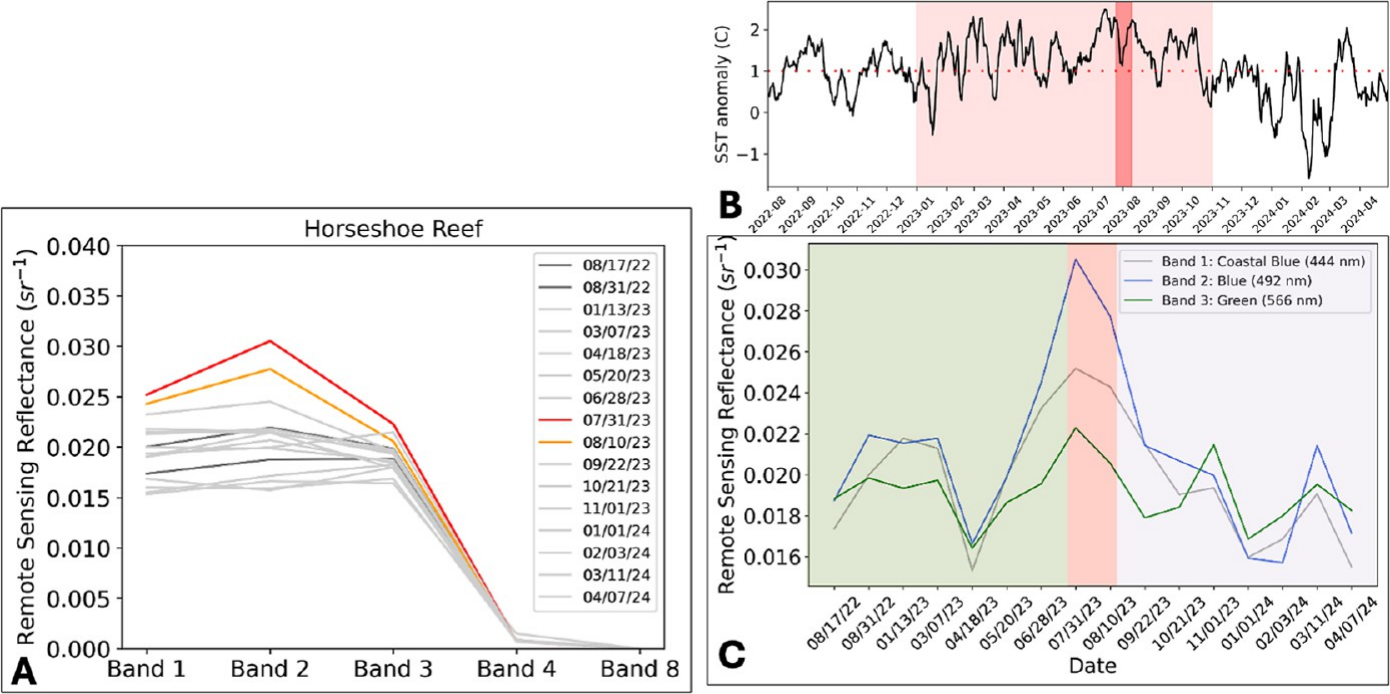
(A) Average normalized remote sensing reflectance (sr^–1^) before, during, and after the bleaching event at Horseshoe Reef. Band 1—444 nm: coastal blue; band 2—492 nm: blue; band 3—566 nm: green; band 4—666 nm: red; and band 8—866 nm: NIR. (B) NOAA daily global sea surface temperature anomaly from August 2022 to April 2024 at Horseshoe Reef. The region highlighted in the lighter red horizontal band shows a prolonged SST anomaly above 1 °C from January to mid October 2023. The region highlighted in the darker red vertical bar is the bleaching period. (C) Time series data displayed in Figure 6A includes only bands 1–3. The green highlighted area is before the bleaching event, the red highlighted area is during the bleaching event, and the purple highlighted area is after the bleaching event.

To ground-truth the fluid lensing data, we compared these results to orthomosaics of Horseshoe Reef.^19^ The large colony in Figure 3 (black box in bottom panel) is the same colony shown in the orthomosaic when healthy (Figure 7A) and bleached (Figure 7B). Further, the fluid lensing images also show the surrounding coral colonies, which appear completely bleached by July 25, 2023 through at least August 8, confirming that we correctly identified the period of bleaching through this novel technique.

**Figure 7:**
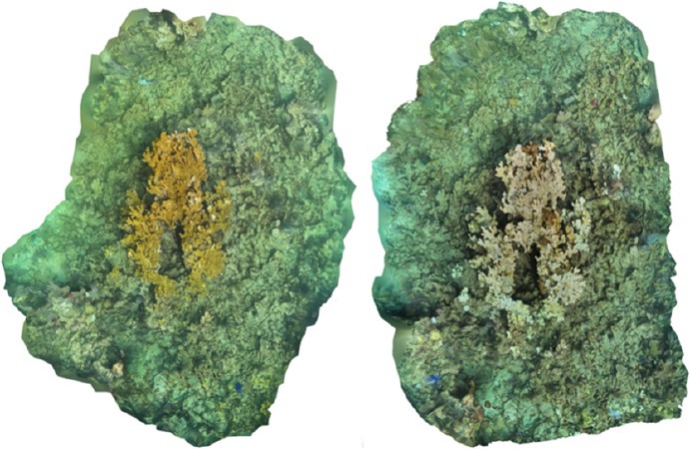
Orthomosaics of healthy colonies on Horseshoe Reef in Key Largo, Florida, from July 11, 2023 (A) and visually bleached colonies from July 25, 2023 (B). Images from^19^.

The SST anomaly from August 2022 to April 2024 at Horseshoe Reef is shown in Figure 6B. From June 14 to September 3 of 2023, the SST anomaly was above 1 °C for 81 days. Figure 6C shows a time series of the same data as Figure 6A only showing bands 1–3. We find that after the bleaching event (highlighted in purple), we see spikes in band 3 in November and March of 2024 (Figure 6C) which may be due to turf algal growth over dead corals on the reef. Anecdotal reports from NOAA found that there was high mortality of at Horseshoe Reef sometime between November 2023 and April 2024.^19^

The average remote sensing reflectance (sr^–1^) for bright sand and shallow water regions are included in Figure S1. We selected random targets (30 × 30 pixels) of sand and water regions near Horseshoe Reef. We found that the bright sand targets show more variability compared to the shallow water regions. Also, the sand pixels have higher reflectance (∼0.03–0.06 across bands 1–3) compared to the bleached pixels (∼0.02–0.03 across bands 1–3).

### Cheeca Rocks Results

Figure 8 shows results from Cheeca Rocks before and during the bleaching event at four reef patches highlighted by red boxes. Radiometric normalization via PIFs is applied to these images. Figure 8A is before the bleaching event and Figure 8B is during the bleaching event. Visually we can see a difference in the normal conditions and bleached corals in the imagery.

**Figure 8:**
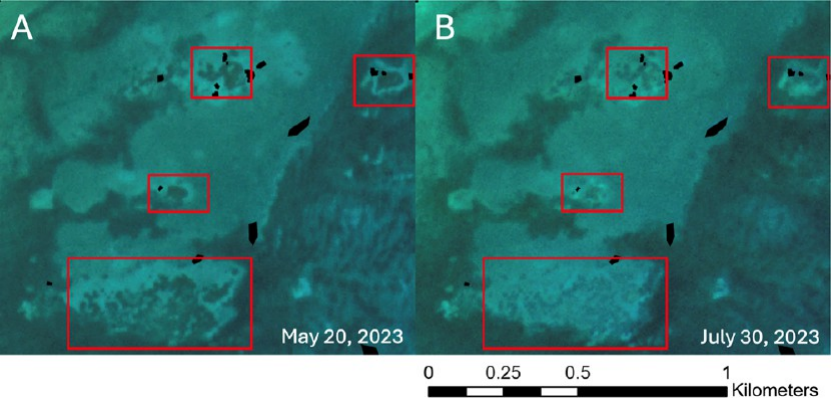
RGB imagery of the SuperDove sensor (PSB.SD) before (left) and during (right) the bleaching event at Cheeca Rocks. Radiometric normalization via PIFs applied to both images. (A) May 20, 2023, is before the bleaching event; (B) July 30, 2023, is during the bleaching event. Red boxes show where there is high coral cover. The red highlighted reef areas lighten significantly during the bleaching event.

The normalized difference between the May 20 and July 30 image is shown in the background in Figure 9. The bright gold regions show the optical signal change that occurs when healthy coral bleaches. C1–C4 are four coral-rich regions at Cheeca Rocks, as demarcated by the red, green, yellow, and pink boxes. Each plot is the average normalized remote sensing reflectance (sr^–1^) before, during, and after the bleaching event from April 2022 to September 2024. The darker lines (gray, blue, and green) are the average of the entire patch, and the lighter lines are the average of the highlighted gold regions within the patch. We found that when we took the average of the entire patch, it did not show a significant peak during the bleaching period (highlighted in red). However, when we selected smaller subsets within the patch (boxes C1–C4) we were able to see a very distinct peak of reflectance, especially in band 2. Before and after the bleaching event (green and purple regions) the data are noisy in all four patches; during the bleaching event (red regions), however, the optical signals move in concert for the three bands. We also see an increase in reflectance after the 2023 bleaching event especially in the Summer of 2024 which could be due to another bleaching or paling event.

**Figure 9:**
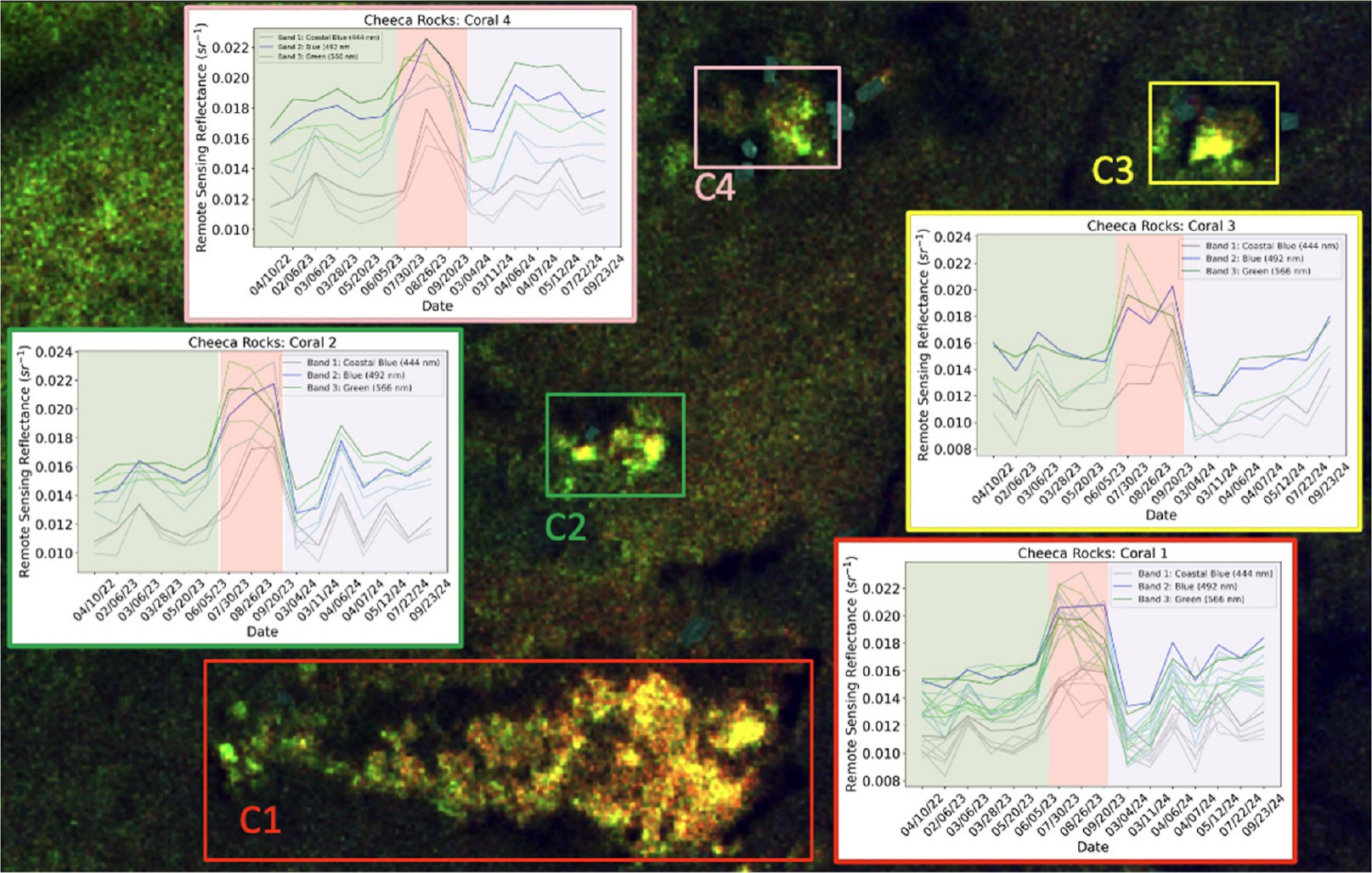
Cheeca Rocks: Background image is the normalized difference between 5/20/23 (see Figure 8A) and 7/30/23 (see Figure 8B) images and the RGB color composite is reversed. The time series of bands 1–3 of the four coral patches before, during, and after the bleaching event are shown for all four smaller regions on the reef (C1 – C4) with high coral cover at Cheeca Rocks. The dark gray, blue, and green lines are the average normalized remote sensing reflectance (sr^–1^) of the entire patch. The lighter lines are the average normalized reflectance of smaller regions within the patch (regions highlighted in bright gold). The green highlights before the bleaching event, red highlights during the bleaching event, and the purple highlights after the bleaching event.

Results of the average remote sensing reflectance of the bright gold regions at each patch for bands 1–8 are located in Figure S2. These results found that band 2 is able to detect the bleaching at each of the four patches. The average remote sensing reflectance (sr^–1^) for bright sand and shallow water regions are included in Figure S3. We selected random targets (30 × 30 pixels) of sand and deep-water regions near Cheeca Rocks. We found that the bright sand targets show more variability compared to the deep-water regions. Also, the sand pixels have higher reflectance (∼0.025–0.5 across bands 1–3) compared to the bleached pixels (∼0.013–0.025 across bands 1–3).

## Coral Bleaching Detection

This study investigates the question: Can we detect coral bleaching using PlanetScope SuperDove imagery in the Florida Keys? Previous studies have used Sentinel 2, Landsat 8, and IKONOS imagery to detect coral bleaching.^10–13,15,17^ These studies have focused mainly on reefs in Australia which have dense coral cover. Our results found that band 2 (492 nm) from the SuperDove imagery is the best indicator of coral bleaching (Figures 6 and 9). This finding is in agreement with^12^ using Sentinel 2A band 2 (490 nm). Due to rapid signal attenuation with depth, previous studies have also found that shallow coral reefs (less than 15 m) are ideal for detection of coral bleaching events. For these reasons, we chose two shallow-water coral reefs, Horseshoe Reef and Cheeca Rocks, for this study. Although we selected clear images with <15% cloud cover and excluded imagery close to storm or rain events, there will inevitably be noise in the data set. We see noise in the time series before and after the bleaching event at both sites (Figures 6C and 9). Noise in the data set could be introduced by several factors, such as colored dissolved organic matter (CDOM), turbidity, high chlorophyll concentrations, or a mixed pixel effect, all of which alter the reflectance patterns of the water.^11^ These effects could also be due to the SuperDove sensor itself, where there is higher noise in the NIR band, which is used for glint correction.^33^ However, because we have in situ field validation (Figures 3 and 7) of the bleaching extent on both Horseshoe Reef and Cheeca Rocks, we can confirm that the elevated reflectance signal is due to bleaching.

Although our study focused on two small reef sites in the Florida Keys, the results suggest that this approach, when combined with in situ observations of bleaching, may be adaptable to other regions. The use of pseudoinvariant features (PIFs) to normalize satellite imagery has been successfully applied in other coral reef environments (e.g., 11; 16; 12; 36), and holds promise for broader application, particularly in clear, shallow waters with available ground-truth data and cloud-free imagery. Unlike current bleaching alerts that rely on SST data to forecast thermal stress, such as those from NOAA’s Coral Reef Watch, our approach directly detects bleaching-related changes in reef reflectance over time (Figures 6 and 9), providing a valuable complement to existing monitoring tools. However, even in the absence of time series information, the “white-out” conditions of a recently bleached, high coral cover reef create an optical signal which is ripe for satellite detection. As satellite resolution and band-with algorithms improve, bleaching signal detection will also improve.

More work needs to be done to test these methods in other regions using SuperDove data. Prior studies typically used Sentinel 2. We utilized the third-generation Planet SuperDove sensor, which has an increased number of bands from four to eight. This increase in the number of bands also closely aligns our SuperDove data with Sentinel 2 data. So far, these methods have only been applied to regions during a severe mass bleaching event where we see >90% bleaching and high coral cover. Horseshoe Reef was a special case in that although coral cover was generally low, there were smaller patches on the reef with very high cover of . When these living coral patches bleached, the signal was easily detectable at our 3 m/pixel resolution. Our study also benefitted from our use of before, during, and after georeferenced optical signals from the airborne fluid lensing data set.

Data availability is also another challenge for detecting coral bleaching from space. Some traditional methods do not include well-georeferenced data nor well-monitored local % coral cover data. Accurate georeferenced information on the presence of healthy (before) and bleached coral (after) populations is critical in trying to decide where to spend limited conservation dollars. Ground-truthing with the NASA airborne fluid lensing data set and the in situ orthomosaics were key to accurate interpretation of coral bleaching on Horseshoe Reef. Our combined method (satellite information and field data) creates a powerful diagnostic and conservation tool. Future work should focus on areas with coral reefs that have (a) high coral cover, (b) coral monitoring programs, and which (c) experience bleaching events that affect >90% of its coral cover.^12^

Coral reefs around the world face an increased risk of bleaching in a changing climate. The need to map live coral cover over time therefore has become more important than ever. This will allow us to know which reefs are healthy or stressed during heatwave events. It will allow us to know which reefs are more resilient than others. A combination of satellite and in situ monitoring will achieve these goals and benefit both ecological research and coral restoration programs in the future. Figure 3 provides a high-resolution example of these combined monitoring methods on Horseshoe Reef.

Airborne fluid lensing missions are ongoing globally as part of the NASA NeMO-Net global coral mapping initiative and freely available at http://nemonet.info. Using NeMO-Net and airborne fluid lensing, coral reef ecosystems are being classified into habitats and monitored at the cm-scale through time. Fluid lensing data are also being used as part of the ongoing NASA MarineVERSE project, which uses the fine-scale, distortion free imagery to train, enhance, and increase the robustness of benthic habitat classification using satellite imagery.^37^

## Supplementary Material



## Notes

Normalized remote sensing reflectance data for bright sand, shallow and deep-water targets (Figures S1 and S3); normalized remote sensing reflectance data extracted from bright gold regions in Figure 9 (Figure S2); image selection tables for Horseshoe Reef and Cheeca Rocks from 2022 to 2024 (Tables S1 and S2) (PDF)
