# Supplementary material for: Impacts of the 2023 Marine Heatwave in the Florida Keys: Detection and Analysis of a Mass Coral Bleaching Event Using Spaceborne Remote Sensing Imagery
Source: Environ Sci Technol. 2025 Jul 21;59(29):15227–35. doi: 10.1021/acs.est.5c03122 (PMC12312086; doi:10.1021/acs.est.5c03122)
Supplement: Supplementary file 1 [file es5c03122_si_001.pdf]

Supporting Information for

Impacts of the 2023 Marine Heatwave in the Florida Keys: Detection and Analysis of a Mass Coral Bleaching Event Using Spaceborne Remote Sensing Imagery

Mariam Ayad[1], Christine M. Lee[2], James W. Porter[3], Ved Chirayath[4], Camilla L. Nivison[3], Kelsey M. Vaughn[3], Raphael Kudela[1].*

[1] Department of Ocean Sciences, University of California, Santa Cruz, Santa Cruz, CA, 95064, USA
[2] Jet Propulsion Laboratory, California Institute of Technology, Pasadena, CA, 91011, USA
[3] Odum School of Ecology, University of Georgia, Athens, GA, 30602, USA
[4] Rosenstiel School of Marine, Atmospheric, and Earth Science, University of Miami, Miami, FL, 33149, USA

Summary information:

Number of pages: 4

Tables: 2

Figures: 3

This file includes:

Supplemental Figure 1. Average normalized remote sensing reflectance (sr^{-1}) for bright sand targets and shallow water regions at Horseshoe Reef. S2

Supplemental Figure 2. Results of the average remote sensing reflectance extracted from the regions in **Figure 9**. S3

Supplemental Figure 3. Average normalized remote sensing reflectance (sr^{-1}) for bright sand targets and deep-water regions at Cheeca Rocks. S4

Supplemental Table 1. Images selected for Horseshoe Reef from 2022-2024. . . . . S4

Supplemental Table 2. Images selected for Cheeca Rocks from 2022-2024. . . . . .S5

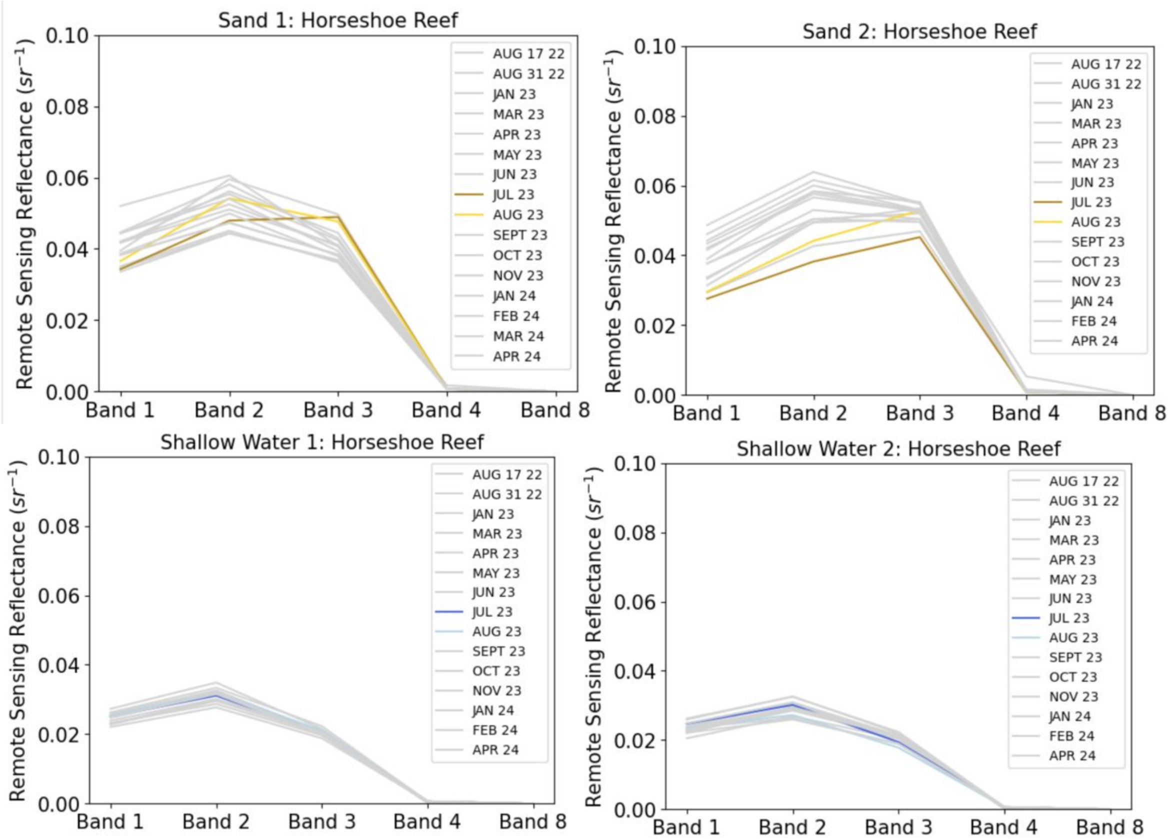

Supplemental Figure 1. Average normalized remote sensing reflectance (sr^{-1}) for bright sand targets (top) and shallow water regions (bottom) at Horseshoe Reef. The highlighted colors in all plots are during the bleaching period.

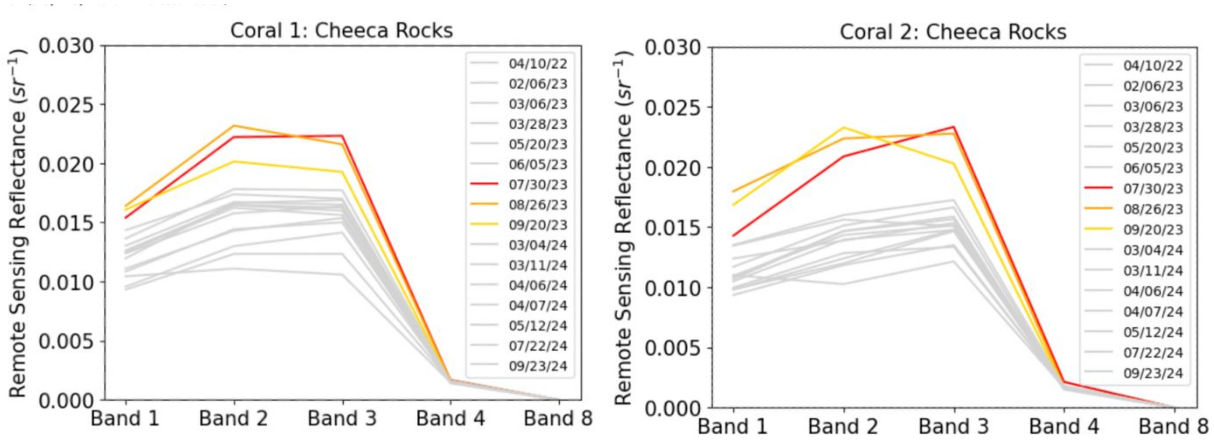

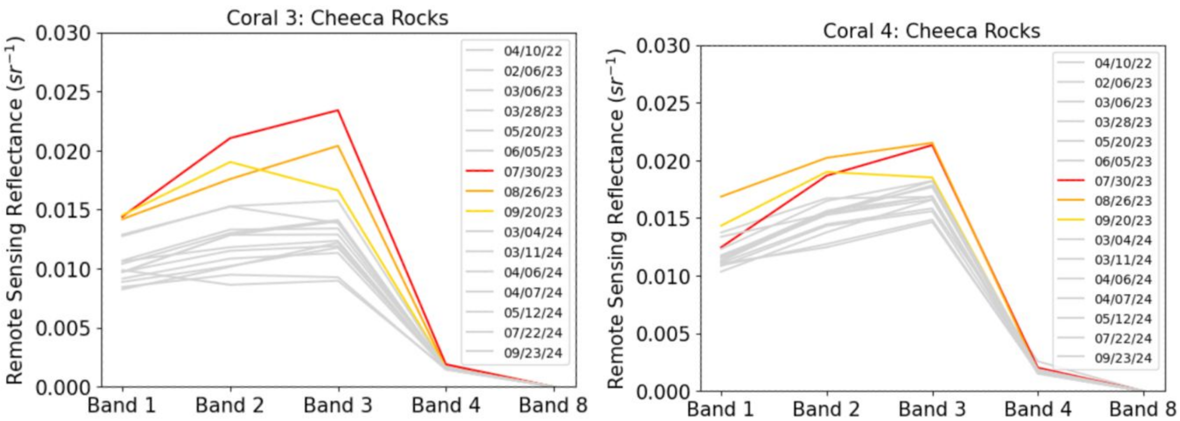

Supplemental Figure 2. Results of the average remote sensing reflectance extracted from the regions in **Figure 9** (highlighted in bright gold). All four coral reef locations show high reflectance in band 2 during the bleaching period (red, orange, and yellow lines) compared to the normal conditions.

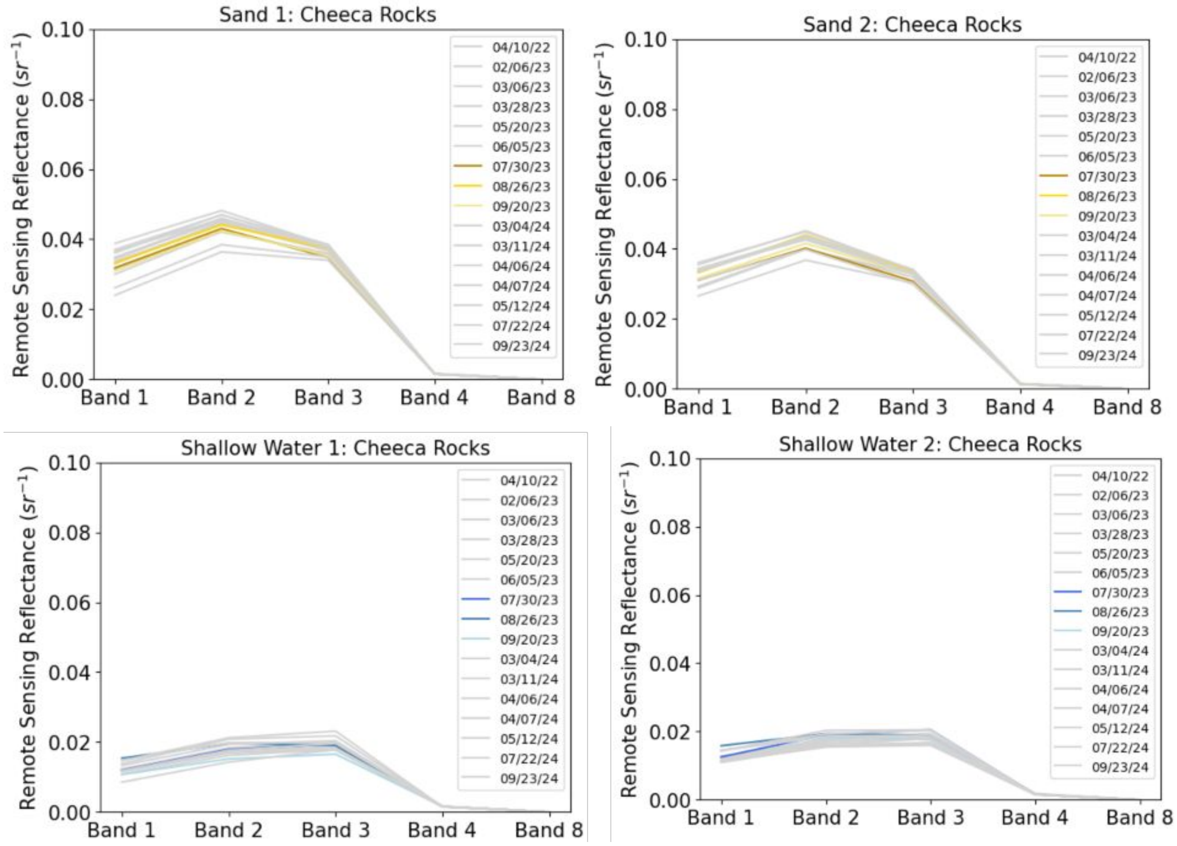

Supplemental Figure 3. Average normalized remote sensing reflectance (sr^{-1}) for bright sand targets (top) and deep water regions (bottom) at Cheeca Rocks. The highlighted colors in all plots are during the bleaching period.

Supplemental Table 1. Images selected for Horseshoe Reef from 2022-2024.

ID	Image date	Solar zenith angle (degrees)	Cloud cover (%)
1	08/17/22	28.4	0
2	08/31/22	37	0
3	01/13/23	53.2	0
4	03/07/23	46.6	0
5	04/18/23	36.7	0
6	05/20/23	30.6	9
7	06/28/23	31.4	12
8	07/31/23	25.2	8
9	08/10/23	25.7	3
10	09/22/23	40.2	2
11	10/21/23	45.8	0
12	11/01/23	48.6	0
13	01/01/24	51.7	0
14	02/03/24	47.8	0
15	03/11/24	43.9	0
16	04/07/24	35.6	5

Supplemental Table 2. Images selected for Cheeca Rocks from 2022-2024.

ID	Image date	Solar zenith angle (degrees)	Cloud cover (%)
1	4/10/22	37	0
2	2/6/23	49.9	0
3	3/6/23	41.4	0
4	3/28/23	40.3	0
5	05/20/23	32.4	0
6	06/05/23	24.9	0
7	07/30/23	33.2	0
8	08/26/23	35.3	0
9	9/20/23	38.7	0
10	3/4/24	38.4	0
11	3/11/24	34.8	0
12	4/6/24	25.6	0
13	4/7/24	35.6	0
14	5/12/24	29.1	0
15	7/22/24	30	0
16	9/23/24	29.1	0
